# Sodium or Not Sodium: Should Its Presence Affect the Accuracy of Pose Prediction in Docking GPCR Antagonists?

**DOI:** 10.3390/ph15030346

**Published:** 2022-03-11

**Authors:** Davide Bassani, Matteo Pavan, Mattia Sturlese, Stefano Moro

**Affiliations:** Molecular Modeling Section (MMS), Department of Pharmaceutical and Pharmacological Sciences, University of Padova, 35131 Padova, Italy; davide.bassani.1@studenti.unipd.it (D.B.); matteo.pavan.7@phd.unipd.it (M.P.); mattia.sturlese@unipd.it (M.S.)

**Keywords:** GPCR, molecular docking, sodium ion, allosteric modulator, antagonist, benchmark, performance, GOLD, PLANTS, Glide

## Abstract

The function of the allosteric sodium ion in stabilizing the inactive form of GPCRs has been extensively described in the past decades. Its presence has been reported to be essential for the binding of antagonist molecules in the orthosteric site of these very important therapeutical targets. Among the GPCR–antagonist crystal structures available, in most cases, the sodium ion could not be experimentally resolved, obliging computational scientists using GPCRs as targets for virtual screening to ask: “Should the sodium ion affect the accuracy of pose prediction in docking GPCR antagonists?” In the present study, we examined the performance of three orthogonal docking programs in the self-docking of GPCR antagonists to try to answer this question. The results of the present work highlight that if the sodium ion is resolved in the crystal structure used as the target, it should also be taken into account during the docking calculations. If the crystallographic studies were not able to resolve the sodium ion then no advantage would be obtained if this is manually inserted in the virtual target. The outcomes of the present analysis are useful for researchers exploiting molecular docking-based virtual screening to efficiently identify novel GPCR antagonists.

## 1. Introduction

G protein-coupled receptors (GPCRs) represent one of the most important protein superfamilies encoded by the human genome. The members of this protein superfamily (more than 800 entities [1]) have been proven to perform a great variety of biological functions in the organism. Among these, very remarkable are the regulation of senses (e.g., smell, taste, gustatory), the regulation of the nervous and immune systems, homeostasis modulation, pain control, and mood balancing [2]. Indeed, it becomes clear why GPCRs are one of the most interesting protein superfamilies for drug discovery, with more than 160 validated drug targets among them [3]. The fact that encourages the scientific community in putting efforts into the research about GPCRs is their huge therapeutic potential. At the present date, about 35% of the FDA-approved drugs are directed towards a GPCR [4,5], and more than 300 molecules are currently in clinical trials, with near one-fifth targeting a novel GPCR protein [4]. These data make clear that the drug discovery research in this field is very active, and much about this superfamily of proteins has yet to be understood.

GPCRs are cataloged into six classes based on their sequence and function similarities: class A (rhodopsin-like receptors), class B (known as “secretin family”), class C (metabotropic glutamate receptors), class D (fungal mating pheromone receptors), class E (cyclic adenosine monophosphate (cAMP) receptors), and class F (frizzled and smoothened receptors) [6]. All GPCRs share a similar organization in their three-dimensional structure; they are membrane protein receptors constituted of a transmembrane domain formed by sevenα-helices (7TM domain), which are linked by three extracellular and three intracellular loops (three ECLs and three ICLs, respectively). The N-terminal (N-ter) domain is located in the extracellular side, while the C-terminal (C-ter) is found intracellularly. The functions of GPCRs are strongly dependent on their conformation and on the changes of this confirmation in time. They exist in an equilibrium between an active and an inactive state [7], and this balance can be shifted upon ligand binding. Indeed, three main families of GPCR ligands have been reported: agonists, antagonists, and inverse agonists. The first group of binders shifts the equilibrium towards the active arrangement of the receptor, while inverse agonists exert the opposite effect, increasing the conformational inactive population and decreasing the GPCR basal activity. Antagonists simply bind to the receptor and prevent the binding of other ligands, without affecting the conformational balance of the GPCR [8].

In drug discovery campaigns aimed to find new molecular entities for GPCR binding, several techniques are used to select, prioritize, and optimize the most promising compounds. Computational tools have acquired a very important role in the latest decades for drug design and discovery, strongly reducing both the time and money required to obtain new drug candidates and elucidating the most important features required to achieve a desired therapeutic effect. The approach chosen from computational medicinal chemistry to reach these ambitious goals depends on the data available about the target of interest. The presence of a three-dimensional structure of the drug target implies the possibility to exploit a structure-based drug design (SBDD) procedure, while its absence prompts the prioritization of the ligand-based drug design (LBDD) techniques. SBDD has proven to be very successful through the pharmaceutical history [9,10], with several campaigns leading to approved drugs or the repositioning of existing drugs on different targets. The most applied technique belonging to the SBDD family is surely “molecular docking” [11]. 

Molecular docking is a computational approach that aims to find the conformation in which a molecule binds to its recognition site, forming a stable complex [12]. Specifically, in the case of drug discovery, the main goal is to elucidate how a ligand (which could be a small molecule, a peptide, or a macromolecule) binds to a biological target of interest (usually a protein or a nucleic acid). Docking algorithms are composed of two main parts: a conformational search algorithm and a scoring function. The first aims to search through the conformational space of the ligand, while the second has the goal of ranking the conformations obtained based on their eligibility for target binding. This fitness evaluation is based on several factors, taking into account different geometrical and energetical parameters. Molecular docking has been successfully applied multiple times for virtual screening (VS) aimed at GPCR drug discovery, both in academic and industrial environments [13]. In these specific cases, attention must be paid to obtain reasonable results from the VS, tuning the docking experiment with respect to both the specific target and the family of ligands considered. A recent study demonstrated that the results of molecular docking on adenosine receptor A_2A_ change if the sodium ion stabilized in the transmembrane domain is considered or not during the calculations [14]. Specifically, that work highlighted a concordance between the computational data and the literature regarding A_2A_ receptor modulation, showing that docking algorithms tend to more efficiently reproduce antagonists’ crystallographic binding modes when the sodium ion also is taken into consideration during the calculations. Indeed, the sodium ion has been reported to be present in the middle of the 7TM region of the receptor in several structures of class A GPCRs, helping stabilize the inactive conformation. The sodium ion, together with its solvation sphere, has been demonstrated to negatively modulate the binding of agonists, without influencing the binding of antagonists [15].

To date, the GPCR group which has prevailed for importance for drug discovery is class A (known as rhodopsin-like receptors), mainly for their centrality in the diseases in which they are involved, as well as for the abundance of resolved structures [16]. These proteins are divided into 19 subfamilies (A1–A19) based on phylogenetic analysis [17], including some receptors which have already become very famous in the pharmaceutical world, such as opioid, adrenergic, histaminergic, cannabinoid, and adenosine receptors. 

Our evaluation starts from the already cited work of Margiotta et al. [14] to explore the influence of the allosteric sodium ion when molecular docking experiments for the diverse class A GPCR antagonists are performed. Indeed, we evaluated the performance of three different and orthogonal docking algorithms (GOLD, Glide, and PLANTS) in reproducing the ligand crystallographic pose of protein–ligand complexes involving an antagonist bound to a class A GPCR. We extended the study to the class A subfamilies of which some antagonist–protein experimental structure is available, also taking into consideration the eventually present complexes involving a reverse agonist bound to the orthosteric binding site.

## 2. Results and Discussion

**Figure 1:**
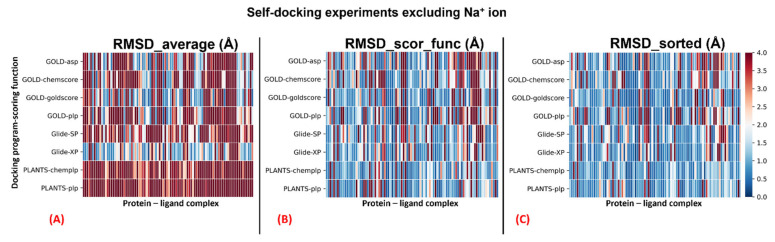
Colormaps show the results of the self-docking calculations not considering the sodium ion within the GPCR receptor 7TM region of the 118 complexes examined in this study. The three plots depict respectively: (**A**) the outcomes derived from the average of the RMSDs of all the poses for each docking run (“RMSD_average”); (**B**) the results obtained just from the RMSD between the crystallographic ligand coordinates and the best-ranked pose from the scoring function (“RMSD_scor_func”); (**C**) the results of the self-docking experiments if just the pose showing the best RMSD value between its coordinates and the crystallographic ones are considered (“RMSD_sorted”). The x-axis enumerates all the different GPCR–antagonist complexes, which are plotted against the different docking program–scoring function pairs used for our study, reported on the y-axis.

**Figure 2:**
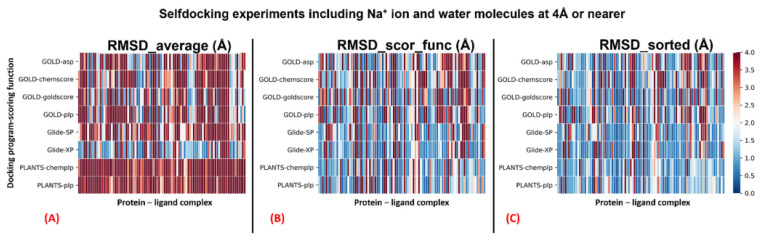
Colormaps show the results of the self-docking calculations executed considering the sodium ion and the water molecules at 4 Å or nearer to it within the GPCR receptor 7TM region of the 118 complexes examined in this study. The three plots depict respectively: (**A**) the outcomes derived from the average of the RMSDs of all the poses for each docking run (“RMSD_average”); (**B**) the results obtained just from the RMSD between the crystallographic ligand coordinates and the best-ranked pose from the scoring function (“RMSD_scor_func”); (**C**) the results of the self-docking experiments if just the pose showing the best RMSD value between its coordinates and the crystallographic ones are considered (“RMSD_sorted”). The x-axis enumerates all the different GPCR–antagonist complexes, which are plotted against the different docking program–scoring function pairs used for our study, reported on the y-axis.

The complete results of the docking runs are reported in the Supplementary Materials (files “Selfdocking_without_sodium.csv” and “Selfdocking_with_sodium.csv”), while a brief per-protocol report is here described by Table 1 and Table 2. A graphical representation of the outcomes of the docking runs is also reported using a colormap representation in Figure 1 and Figure 2. In these plots, the colorimetric scale delineating the RMSD values starts from 0 Å, corresponding to a docking pose perfectly superposable to the crystallographic one (maximum docking performance, represented by the dark blue color), and reaches values of 4 Å or higher (minimum docking performance, all represented by the dark red color), which stands for a very suboptimal overlay between the coordinates of the pose produced and the ones of the crystallographic conformation. The results have been reported using three different metrics: “RMSD_average”, which represents the mean RMSD of all the poses obtained; “RMSD_scor_func”, which is the average value of the RMSDs obtained by the poses which were top-ranked by the scoring functions in each docking run; and “RMSD_sorted”, which represents the mean value of the RMSDs obtained from the poses with the lowest RMSD value in each docking calculation. As mentioned, the analysis of the results has also been executed on each docking program–scoring function pair exploited in the study (Table 1 and Table 2). Moreover, to better inspect the effect of the sodium ion in the docking simulations, the analysis has also been applied to separate the group of proteins in which the sodium ion is present in the crystallographic structure considered (26 systems) from the other entries (92 complexes). The per-protocol inspections of these last results are reported in the Supplementary Materials (Tables S2–S5). 

The outcomes of our experiment highlight how all the algorithms used show an overall good performance in GPCR–antagonist self-docking. Among the others, the pairs “Glide-SP”, “PLANTS_CHEMPLP_”, and “PLANTS_PLP_” were always able to produce an “RMSD_sorted” value of less than 2 Å with respect to the crystallographic coordinates. Even if the scoring functions allowed obtaining reasonable RMSD values (as observable from the “RMSD_scor_func” columns in Table 1 and Table 2), the solutions are given by them rarely corresponded to the ones with the lowest RMSD. As expected, an ant colony optimization algorithm such as PLANTS tends to produce poses with less three-dimensional conservation compared to a genetic algorithm such as GOLD or a systematic method such as Glide, and this is evidenced by the higher values of “RMSD_average” given by both docking program–scoring function pairs involving PLANTS. On the other hand, the higher variability in the poses produced could be the reason for the fact that PLANTS can obtain solutions with very low RMSD, as demonstrated by the “RMSD_sorted” results, which are far below 2 Å in all the cases reported in this study (also when the complexes are separated based on the presence of the sodium ion in the original PDB structure, as depicted in Supplementary Materials, Tables S2–S5). GOLD and Glide both performed remarkably, with “goldscore” giving the best results among the scoring functions implemented for GOLD in all the metrics used for the analysis (exception made for the “RMSD_scor_func” value when considering the sodium ion and the water molecules at 4 Å or nearer to it in the calculations). Comparing “Glide-SP” and “Glide-XP” outcomes, even if the first can obtain lower “RMSD_sorted” values, is important to notice that the XP protocol is the overall best performing when considering the “RMSD_average”, always giving a value below 3 Å for this parameter. The choice between the two for GPCRantagonist virtual screening (VS) should so be based on the specific case examined. Indeed, “Glide-SP” would be more beneficial in the VS of a GPCR antagonist with already known scaffold and properties (eventually coming from “focused libraries”), while “Glide-XP” would be more effective when a library with molecules characterized by higher diversity is taken into account. When considering the use of “Glide-XP” instead of “Glide-SP” for large VS of high-diversity entities for GPCR antagonism, the medicinal chemists should always consider the higher computational times required for the XP function (passing from the 10 s/compound of “Glide-SP” to about 2 min/compound of “Glide-XP”, as reported on the developer’s page [18]). 

**Figure 3:**
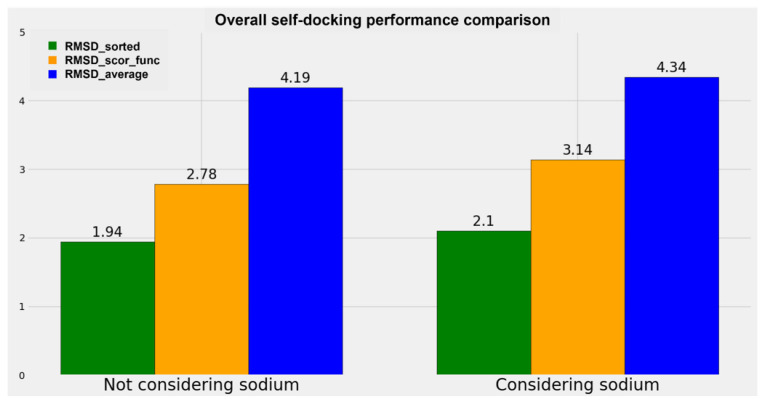
Plot representing the comparison of the overall performance of the different docking algorithms implemented in this study when the sodium ion is not considered during the calculation (on the **left**) and when both the sodium ion and the crystal water molecules at 4 Å or nearer to it are included (on the **right**). The metrics used for the comparison are the “RMSD_average”, the “RMSD_scor_func”, and the “RMSD_sorted” values already described in the present study.

**Figure 4:**
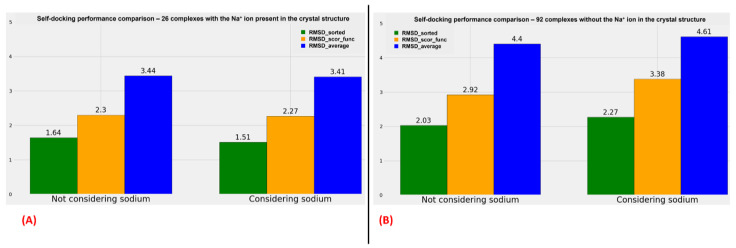
(**A**) Plot representing the comparison of the overall performance of the different docking algorithms implemented in this study when the sodium ion is not considered during the calculation (on the **left**) and when both the sodium ion and the crystal water molecules at 4 Å or nearer to it are included (on the **right**), focusing just on the 26 GPCR–antagonist complexes in which the sodium ionis already present in the crystal structure. (**B**) Graphical representation of the comparison of the overall performance of the different docking algorithms used in this study when the sodium ionis not considered during the calculation (on the **left**) and when both the sodium ion and the crystal water molecules at 4 Å or nearer to it are included (on the **right**), focusing just on the 92 GPCR–antagonist complexes in which the sodium ionis not present on the original crystal structure.

A graphical representation of the comparison between the performance of the algorithms when the sodium ions are considered or not is reported in Figure 3, while two analog diagrams are reported in Figure 4 (based on Tables S2–S5, which can be examined in the Supplementary Materials) to give a more immediate visualization of the outcomes divided based on whether the sodium ion is present in the original crystallographic complexes.

The results obtained show a small decrease in all the metrics used when the sodium ion is not considered in the docking runs. Specifically, the decrement in “RMSD_average” is 3.46%, the reduction in “RMSD_scor_func” is 11.47% (this higher value has to be attributed to the scoring functions), and the diminution in “RMSD_sorted” is 7.62%. Considering the decreases in the order of magnitude of the RMSD of the docking results (which is around the very promising value of 2 Å for the best pose obtained and around 3 Å for the best solution given by the scoring functions), we can conclude that no big difference in the docking performance regarding a GPCR–antagonist system is achieved if the sodium ion is taken into account during the calculation. 

The metrics used for the comparisons are the “RMSD_average”, the “RMSD_scor_func”, and the “RMSD_sorted” values already described in the present study. The overall figure is useful to compare the performance of the docking algorithms when the sodium is present in the original crystal structure and when it is not.

With an examination of the data coming from Tables S2–S5, of which the comparison of the overall results is plotted in Figure 4, we can see an analog trend of the outcomes when sodium is considered or not during the docking runs. It is interesting to notice that when the sodium is already present in the crystal structure, the RMSD values obtained from the docking poses tend to be more promising, but this has to be weighed on the fact that, in that case, the exact position of the sodium is known, and so the possible error coming from the manual placing of this alkaline ion in the 7TM region is removed. Moreover, a comparison should be made very carefully when data coming from only 26 complexes (the ones having the sodium crystallographic ally resolved) are juxtaposed to the ones derived from a larger set of 92 structures (the complexes in which the sodium ion is missing in the crystal structure).

On the contrary, important information is obtainable if the comparison is limited between the two groups of proteins. Indeed, as shown in Figure 4 (as well as Tables S2–S5), if the sodium ions are already present in the GPCR–antagonist crystallographic complex, no relevant difference can be noticed between the results coming from the docking runs in which the alkaline ion is considered and the ones derived from the calculation in which also sodium and the water molecules surrounding it are taken into account. Furthermore, the analysis shows a very slight decrease in the RMSD values when the crystallographic sodium and the water molecules at 4 Å or nearer to it are retained during the calculations.

On the other side, more important changes in the RMSD metrics used are highlightable comparing the outcomes of the docking runs for the complexes in which the sodium ion is not present in the crystal structure. In this case, all “RMSD_average”, “RMSD_scor_func”, and “RMSD_sorted” values show an increase if the alkaline ion with its surrounding water molecules is inserted in the complex and considered during the calculation. 

The results of our study show that when performing molecular docking experiments on GPCR antagonists, the sodium ion present in the allosteric 7TM pocket should be considered during the calculation only if it is already present in the crystal structure used as the protein target. If the GPCR on which the research is based does not have antagonist-bound crystallographic structures in which the sodium ion is present, any advantage will be obtained if it is manually placed in its allosteric pocket, and so the execution of the docking calculations without this alkaline ion should be considered. A possible reason for this behavior could be related to the fact that the small benefit coming from taking into account the sodium ion when performing the virtual screening would be demolished by the inevitable error coming from the manual placing of this ion in its allosteric pocket. We also assert that this type of uncertainty would not be canceled even if advanced computational approaches would be used for sodium placement, because of the errors that these techniques inexorably bring with them.

**Figure 5:**
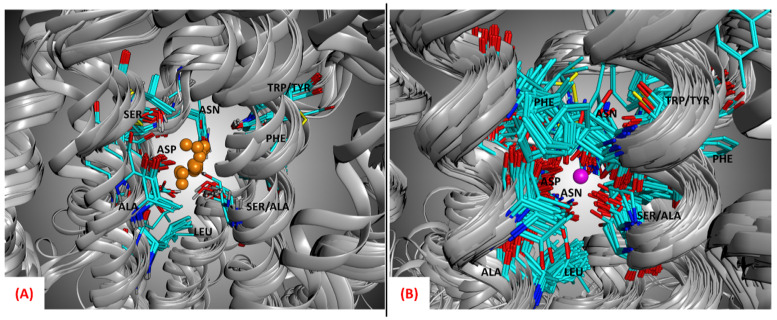
(**A**) Representation of the allosteric sodium binding site of all the 26 GPCR–antagonist complexes considered in this study which had the sodium ion present in their crystallographic structures. As depicted, the position of the sodium (the orange atoms in the image) is well conserved, as are the type and orientation of the side chains of the amino acids surrounding it, which help stabilize the alkaline ion. (**B**) Representation of the allosteric sodium binding site of all the 92 GPCR–antagonist complexes considered in this study in which the sodium ion is not present in the crystallographic structures. As can be seen, the potential position of the sodium (the purple atom in the image, which comes from the crystal upon which all proteins have been superposed, PDB code: 5IU4) is well conserved, as are the type and orientation of the side chains of the amino acids surrounding it, which help to stabilize the alkaline ion in its 7TM allosteric pocket.

The importance of the allosteric sodium ion for the binding of antagonists to class A GPCRs has been extensively described in the literature [15,19]. Moreover, as observable from Figure 5, the side chains of the amino acids located in the allosteric sodium binding site are conservatively orientated towards the alkaline ion location even if all the structures represented do not have the sodium ion present in the crystallographic complex, showing that this alkaline ion has to be present in its site to guarantee the antagonist activity. 

Molecular docking techniques are known for not being able to distinguish agonism from antagonism. Indeed, this family of computational approaches has the goal of highlighting the potential binders for a target, but their results cannot be related to a specific type of outcomes that this binding will have on the target itself (which has to be evaluated by the medicinal chemistry experts, based on their expertise and the communication with other professionals of the pharmaceutical world). These limitations of the technique of molecular docking may be the reason for the very low difference between the results coming from the cases in which sodium is considered or not in the calculations. 

## 3. Materials and Methods

For each of the 19 GPCR class A subfamilies, the crystal structures available in the Protein Data Bank [20] (PDB, latest access 15 January 2022) were inspected. Each entry with a human GPCR protein complexed with a small molecule orthosteric antagonist crystallized together was selected to build the starting database of our study. If multiple crystals of a protein bound to the same ligand existed, only the highest resolution crystal with the sodium ion present was selected. To give a more comprehensive panoramic of the role of allosteric sodium in GPCRbinding, the structures with an inverse agonist were also considered for this study (e.g., 6K1Q, 7F83, 7B6W, 7BVQ). In the end, 118 protein–ligand complexes involving a GPCR and a small molecule antagonist were obtained (a comprehensive list is reported in the Supplementary Materials, Table S1).

The 118 complexes were downloaded from the PDB and imported into Molecular Operating Environment (MOE) suite [21], the main molecular modeling program that we used in this work. Each system was then prepared with a protocol involving the tools included in the MOE package. First, the “Structure Preparation” program was used to rebuild the small missing loops in the structures and to adequately select the orientation of alternate crystallographic residues based on occupancy. Then, the most proper protonation state for each amino acid was determined with the “Protonate 3D” tool, setting 7.4 as the pH value for the environment. Subsequently, the added hydrogen atoms were minimized with the AMBER10:EHT [22] force field implemented in MOE. Finally, each non-protein, non-ligand, and non-sodium molecule was deleted from the systems, with an exception made for the water molecules solvating the sodium ion (weused 4 Å as the cut-off radius), when present. 

The systems were then separated based on whether they had the sodium ion crystallized in their original PDB structure. Among all the complexes downloaded, 26 already had the sodium present in the crystal, while 92 did not (the distinction is highlighted in the Supplementary Materials, Table S1). All the systems in which the sodium was not present were properly treated, inserting the sodium ion with its solvation water molecules. The position of the sodium and the water molecules was chosen according to the PDB crystal 5IU4, the complex with the best resolution, R-value, and R-free value balance among all the entries considered. This choice was also supported by the fact that when superposing all the 7TM regions of the protein–ligand systems with the sodium crystallized, the position of this alkaline ion is very conservative, as observable from Figure 5 (the average RMSD between the coordinates of the sodium ions and the sodium ion of the reference structure 5IU4 was calculated to be 0.75Å). It is important to mention that the crystal 5IU4 was not considered for the docking calculations because, even if its resolution is optimal, it is significantly mutated in the 7TM region. Another ADORA2 crystal structure bearing the same ligand (ZMA), 6LPJ, shows a very similar resolution (1.80Åversus the 1.72 Å of 5IU4) and does not show mutations in the 7TM domain.

Our self-docking approach consisted of the separation of each ligand from its crystallographic GPCR structure, its preparation, and its molecular docking inside the orthosteric binding site with three different orthogonal programs, namely GOLD [23] (based on a genetic algorithm, developed and licensed by CCDC), Glide [24] (a systematic docking program developed and distributed by Schrödinger), and PLANTS [25] (an ant colony optimization algorithm developed by the University of Tübingen). For each of the programs, all the scoring functions supported were used. Specifically, GOLD was used in four different parallel runs, applying the scoring functions “goldscore”, “chemscore”, “asp”, and “plp”. The two Glide calculations for each ligand were executed first with “Glide-SP” and then with “Glide-XP”, while the docking runs with PLANTS exploited the scoring functions “PLANTS_CHEMPLP_” and “PLANTS_PLP_”. For each program–scoring function pair, five poses were produced for each ligand, and each of those was compared with the crystallographic pose to calculate the root-mean-square deviation (RMSD) between the coordinates of the two conformations.

This whole procedure was executed twice, first setting the docking programs to not consider the sodium ion and the water molecules solvating it and then setting the programs to take into account both sodium and the water molecules placed at 4 Å or nearer to the alkaline ion. 

## 4. Conclusions

In the present study, we examined the effect of considering the allosteric sodium ion when molecular docking approaches are applied to GPCR antagonists. To accomplish our task, we collected 118 GPCR–antagonist complexes, both with and without the sodium ion present in the crystallographic structure. For the systems in which this alkaline ion was not present, a manual insertion of the sodium and its surrounding water molecules was executed based on superposition with a very high-resolution structure (PDB: 5IU4), after having established that the position of the ion is very conservative in the GPCR–antagonist crystals. Then, we executed self-docking experiments of the orthosteric GPCR ligands with three orthogonal docking programs (GOLD, Glide, and PLANTS) both considering and not considering the sodium ion and its surrounding water molecules. What emerged from the present work is the finding that the performance of the docking programs (enucleated in three different metrics, “RMSD_average”, “RMSD_sorted”, and “RMSD_scor_func”) does not significantly change between the two cited scenarios. Going deeper into the analysis of the results, we highlighted that a small increment in the docking programs’ performance is observable if the sodium ion is kept during the docking runs just for those crystal structures in which the alkaline ion was resolved, while for the other complexes the trend is the opposite, favoring the solution of not considering sodium during the docking calculations. The outcomes of the present work are helpful to increase the knowledge about the performance of docking programs when applied to research about GPCR antagonists, and we are confident that the pharmaceutical experts that are putting effort into this fascinating field will benefit from our work.

## Figures and Tables

**Table 1:** Table showing the results of the self-docking calculations executed without considering the sodium ion.

Self-Docking Results—Na^+^ and H_2_O Not Considered			
	RMSD_Average (Å)	RMSD_Scor_Func (Å)	RMSD_Sorted (Å)
GOLD-goldscore	3.60	2.83	1.86
GOLD-chemscore	4.45	3.25	2.46
GOLD-asp	3.87	2.91	2.14
GOLD-plp	4.60	3.48	2.56
Glide-SP	4.16	2.57	1.73
Glide-XP	2.67	2.46	1.89
PLANTS_CHEMPLP_	4.96	2.12	1.35
PLANTS_PLP_	5.18	2.58	1.54
**All the molecular docking experiments**	**4.19**	**2.78**	**1.94**

**Table 2:** Table showing the results of the self-docking calculations executed considering the sodium ionand the water molecules surrounding it.

Self-Docking Results—Na^+^ and H_2_O Placed at 4 Å or Nearer to It Both Considered			
	RMSD_Average (Å)	RMSD_Scor_Func (Å)	RMSD_Sorted (Å)
GOLD-goldscore	4.07	3.93	2.33
GOLD-chemscore	4.53	3.90	2.82
GOLD-asp	4.13	3.25	2.40
GOLD-plp	4.52	3.51	2.56
Glide-SP	4.40	2.55	1.67
Glide-XP	2.81	2.62	1.90
PLANTS_CHEMPLP_	5.16	2.60	1.49
PLANTS_PLP_	5.14	2.76	1.62
**All the molecular docking experiments**	**4.34**	**3.14**	**2.10**

## Data Availability

Data is contained within the article and Supplementary Material.

## Supplementary Materials

The following are available online at https://www.mdpi.com/article/10.3390/ph15030346/s1. Table S1. Complete list of all the 118 GPCR-antagonist complexes used for this study. For each system, the class A subfamily, the PDB code, the encoding gene, and the presence of the allosteric sodium ion in the structure are annotated. Table S2. Table showing the results of the self-docking calculations executed without considering the sodium ion during the calculations. This chart represents exclusively the 26 GPCR-antagonist complexes in which the sodium ion is present in the crystal structure. Table S3. Table showing the results of the self-docking calculations executed taking into account the sodium ion and the water molecules at 4 Å or nearer to it. This chart represents exclusively the 26 GPCR-antagonist complexes in which the sodium ion is present in the crystal structure. Table S4. Table showing the results of the self-docking calculations executed without considering the sodium ion during the calculations. This chart represents exclusively the 92 GPCR-antagonist complexes in which the sodium ion is not present in the crystal structure. Table S5. Table showing the results of the self-docking calculations executed taking into account the sodium ion and the water molecules at 4 Å or nearer to it. This chart represents exclusively the 92 GPCR-antagonist complexes in which the sodium ion is not present in the crystal structure.
