# Supplementary material for: Sodium or Not Sodium: Should Its Presence Affect the Accuracy of Pose Prediction in Docking GPCR Antagonists?
Source: Pharmaceuticals (Basel). 2022 Mar 11;15(3):346. doi: 10.3390/ph15030346 (PMC8950631; doi:10.3390/ph15030346)
Supplement: Supplementary file 1 [file pharmaceuticals-15-00346-s001.zip › pharmaceuticals-1611922-supplementary/Supplementary_Material.pdf]

Table S1. Complete list of all the 118 GPCR-antagonist complexes used for this study. For each system, the class A subfamily, the PDB code, the encoding gene, and the presence of the allosteric sodium ion in the structure are annotated.

GPCR Subfamily	PDB code	GPCR receptor gene	Na^+ crystallized
A01	4MBS	CCR5	NO
A01	6AKX	CCR5	NO
A01	6AKY	CCR5	NO
A02	30DU	CXCR4	NO
A03	4YAY	AGTRL1	NO
A03	4ZUD	AGTRL1	NO
A04	4DJH	OPRK1	NO
A04	4N6H	OPRM1	YES
A05	4EA3	OPRL1	NO
A05	5DHG	OPRL1	NO
A05	5DHH	OPRL1	NO
A05	6RZ4	CYSLTR1	YES
A05	6RZ5	CYSLTR1	YES
A05	6RZ7	CYSLTR2	NO
A05	6RZ8	CYSLTR2	NO
A05	6RZ9	CYSLTR2	NO
A05	7K15	LTB4R	YES
A06	4S0V	HCRTR2	NO
A06	4ZJ8	HCRTR1	NO
A06	4ZJC	HCRTR1	NO
A06	5WQC	HCRTR2	NO
A06	6TO7	HCRTR1	YES
A06	6TOD	HCRTR1	YES
A06	6TOS	HCRTR1	YES
A06	6TOT	HCRTR1	NO
A06	6TP3	HCRTR1	NO
A06	6TP4	HCRTR1	NO
A06	6TP6	HCRTR1	NO
A06	6TPN	CCKBR	NO
A06	6TQ4	HCRTR1	YES
A06	6TQ6	HCRTR1	NO
A06	6TQ7	HCRTR1	NO
A06	6TQ9	HCRTR1	NO
A06	6V9S	HCRTR1	NO
A06	7F8U	CCKAR	NO
A06	7F8Y	CCKAR	NO
A07	5X93	EDNRB	NO
A07	5XPR	EDNRB	NO
A07	6K1Q	EDNRB	NO
A07	7F83	GHSR	NO
A09	5ZBH	NPY1R	NO
A09	5ZBQ	NPY1R	NO
A09	6E59	TACR1	NO
A09	6HLL	TACR1	NO
A09	6HLO	TACR1	NO
A09	6HLP	TACR1	NO
A09	7DDZ	NPY2R	NO
A11	4XNW	P2RY1	NO

A12	4NTJ	P2RY1	NO
A12	5ZKP	PTAFR	NO
A13	3V2Y	S1PR1	NO
A13	4Z34	LPAR1	NO
A13	4Z35	LPAR1	NO
A13	4Z36	LPAR1	NO
A13	5TGZ	CNR1	NO
A13	5U09	CNR1	NO
A13	5ZTY	CNR2	NO
A14	5YHL	PTGER2	NO
A14	5YWY	PTGER2	NO
A14	6D26	PTGDR2	NO
A14	6D27	PTGDR2	NO
A14	6IIU	TBXA2R	NO
A14	6IIV	TBXA2R	NO
A15	3VW7	F2R	YES
A15	5NDD	F2RL1	YES
A17	2RH1	ADRB2	NO
A17	3D4S	ADRB2	NO
A17	3NY9	ADRB2	NO
A17	3PBL	DRD2	NO
A17	6A93	HTR2A	NO
A17	6A94	HTR2A	NO
A17	6CM4	DRD2	NO
A17	6DRX	HTR2B	NO
A17	6DRZ	HTR2B	NO
A17	6DS0	HTR2B	NO
A17	6E67	ADRB2	NO
A17	6IQL	DRD4	NO
A17	6KUX	ADRA2A	NO
A17	6LUQ	DRD2	NO
A17	6PS2	ADRB2	NO
A17	6PS3	ADRB2	NO
A17	6PS4	ADRB2	NO
A17	6PS5	ADRB2	NO
A17	6PS6	ADRB2	NO
A17	6WH4	HTR2A	NO
A17	7B6W	ADRA1B	NO
A17	7BVQ	ADRB1	NO (not in the allosteric pocket)
A17	7DFP	DRD2	NO
A17	7VOD	HTR2A	NO
A17	7VOE	HTR2A	NO
A18	3REY	ADORA2A	NO
A18	3RZE	HRH1	NO
A18	3UZA	ADORA2A	NO
A18	5CXV	CHRM1	NO
A18	5DSG	CHRM4	NO
A18	5IU7	ADORA2A	YES
A18	5IU8	ADORA2A	YES
A18	5IUA	ADORA2A	YES
A18	5IUB	ADORA2A	YES

A18	5MZJ	ADORA2A	YES
A18	5MZP	ADORA2A	YES
A18	5N2S	ADORA1	NO
A18	5OLH	ADORA2A	YES
A18	5OLO	ADORA2A	YES
A18	5OLV	ADORA2A	YES
A18	5OLZ	ADORA2A	YES
A18	5UIG	ADORA2A	NO
A18	5ZK3	CHRM2	NO
A18	5ZKB	CHRM2	NO
A18	5ZKC	CHRM2	NO
A18	6GT3	ADORA2A	YES
A18	6LPJ	ADORA2A	YES
A18	6OL9	CHRM5	NO
A18	6WQA	ADORA2A	YES
A18	6ZDR	ADORA2A	YES
A18	6ZDV	ADORA2A	YES
A18	7ARO	ADORA2A	YES
A19	5V54	HTR1B	NO

Table S2. Table showing the results of the self-docking calculations executed without considering the sodium ion during the calculations. This chart represents exclusively the 26 GPCR-antagonist complexes in which the sodium ion is present in the crystal structure.

**Self-docking results – Na^+ and H_2O not considered – data representing only the 26 complexes having the sodium ion crystallized**			
	**RMSD_average (Å)**	**RMSD_scor_func (Å)**	**RMSD_sorted (Å)**
GOLD-goldscore	2.38	2.27	1.36
GOLD-chemscore	3.77	3.27	2.37
GOLD-asp	2.88	2.30	1.95
GOLD-plp	3.77	2.82	2.17
Glide-SP	3.64	2.31	1.61
Glide-XP	2.31	2.28	1.48
$PLANTS_{CHEMPLP}$	4.14	1.26	0.96
$PLANTS_{PLP}$	4.66	1.86	1.26
**All the molecular docking experiments**	**3.44**	**2.30**	**1.64**

Table S3. Table showing the results of the self-docking calculations executed taking into account the sodium ion and the water molecules at 4 Å or nearer to it. This chart represents exclusively the 26 GPCR-antagonist complexes in which the sodium ion is present in the crystal structure.

**Self-docking results – Na^+ and H_2O placed at 4 Å or nearer to it both considered – data representing only the 26 complexes having the sodium ion crystallized**			
	**RMSD_average (Å)**	**RMSD_scor_func (Å)**	**RMSD_sorted (Å)**
GOLD-goldscore	2.69	2.80	1.16
GOLD-chemscore	3.75	3.34	2.48
GOLD-asp	3.00	2.85	2.07
GOLD-plp	3.21	1.85	1.77
Glide-SP	4.09	1.62	1.12
Glide-XP	1.58	1.56	1.11
$PLANTS_{CHEMPLP}$	4.40	1.99	1.12
$PLANTS_{PLP}$	4.52	2.18	1.23
**All the molecular docking experiments**	**3.41**	**2.27**	**1.51**

Table S4. Table showing the results of the self-docking calculations executed without considering the sodium ion during the calculations. This chart represents exclusively the 92 GPCR-antagonist complexes in which the sodium ion is not present in the crystal structure.

Self-docking results – Na^+ and H_2O not considered – data representing only the 92 complexes that don't have the sodium ion crystallized			
	**RMSD_average (Å)**	**RMSD_scor_func (Å)**	**RMSD_sorted (Å)**
GOLD-goldscore	3.95	2.99	2.01
GOLD-chemscore	4.65	3.34	2.49
GOLD-asp	4.15	3.08	2.19
GOLD-plp	4.84	3.67	2.67
Glide-SP	4.31	2.64	1.77
Glide-XP	2.77	2.51	2.00
$PLANTS_{CHEMPLP}$	5.18	2.36	1.46
$PLANTS_{PLP}$	5.32	2.78	1.63
**All the molecular docking experiments**	**4.40**	**2.92**	**2.03**

Table S5. Table showing the results of the self-docking calculations executed taking into account the sodium ion and the water molecules at 4 Å or nearer to it. This chart represents exclusively the 92 GPCR-antagonist complexes in which the sodium ion is not present in the crystal structure.

Self-docking results – Na^+ and H_2O placed at 4 Å or nearer to it both considered - data representing only the 92 complexes that don't have the sodium ion crystallized			
	**RMSD_average (Å)**	**RMSD_scor_func (Å)**	**RMSD_sorted (Å)**
GOLD-goldscore	4.46	4.25	2.66
GOLD-chemscore	4.76	4.06	2.92
GOLD-asp	4.45	3.37	2.50
GOLD-plp	4.89	3.98	2.78
Glide-SP	4.49	2.81	1.82
Glide-XP	3.15	2.91	2.12
$PLANTS_{CHEMPLP}$	5.36	2.77	1.60
$PLANTS_{PLP}$	5.32	2.92	1.73
**All the molecular docking experiments**	**4.61**	**3.38**	**2.27**
